# The True Value of Quantitative Imaging for Adrenal Mass Characterization: Reality or Possibility?

**DOI:** 10.3390/cancers15020522

**Published:** 2023-01-14

**Authors:** Arnaldo Stanzione, Valeria Romeo, Simone Maurea

**Affiliations:** Department of Advanced Biomedical Sciences, University of Naples “Federico II”, 80131 Naples, Italy

The widespread use of cross-sectional imaging modalities, such as computed tomography (CT) and magnetic resonance imaging (MRI), in the evaluation of abdominal disorders has significantly increased the number of incidentally detected adrenal abnormalities, particularly adrenal masses [1]. In this clinical scenario, the main goal is the differential diagnosis between benign and malignant lesions in order to select the most appropriate management option (e.g., follow-up or further investigations) for each patient. Alongside imaging, clinical and laboratory assessments of adrenal function allow for the classification of adrenal tumors as hypersecreting and, thus, for diagnosing specific disorders [2]. Conversely, an adrenal mass may not be associated with abnormal hormone hypersecretion or it may produce nonfunctional agents, being classified as non-hypersecreting. In these patients, tumor characterization is fundamental, and imaging plays a crucial role [3]. CT and MRI provide detailed anatomic features of adrenal masses and may also demonstrate presumptive imaging criteria for tissue characterization. Furthermore, using specific radiopharmaceuticals’ different nuclear imaging techniques can offer additional functional evaluation for problem solving and differential diagnoses of adrenal masses indeterminate through CT and MRI.

In a review recently published in *Cancers*, Barat et al. [4] comprehensively illustrate the role of different imaging modalities in the characterization of adrenal masses, presenting the principles and techniques behind conventional imaging assessment as well as the potential added value of novel quantitative approaches. Interestingly, they remind us of how quantitative approaches are already a meaningful component of the radiological routine [5]. Two main examples are the assessment of interval growth through maximum diameter measurement on baseline and follow-up imaging in addition to the calculation of the mean attenuation value by using regions of interest through CT. Indeed, a growth rate of >5 mm per year is suspicious for malignancy, while Hounsfield units allow for the quantitative confirmation of the presence of microscopic fat within adrenal masses, which is sufficient for accurately identifying lipid-rich adenomas. On the other hand, chemical shift MRIs could also provide quantitative data, but without an additional benefit in terms of diagnostic accuracy over the less time-consuming visual assessment of this sequence [6]. Of note, adrenal adenomas with poor fat contents can also be correctly diagnosed with a quantitative approach; however, this requires the administration of contrast agents and a dedicated imaging protocol to compute the wash-out (this can be done through either CT or MRI) [7]. Despite all of these possibilities, a relatively low percentage of all adrenal masses still remains indeterminate at cross-sectional imaging.

The new kid on the adrenal quantitative imaging block is radiomics, and the authors have concisely but wisely introduced readers to this vast topic, summarizing the existing evidence while underlining current challenges [8]. The promise of radiomics is to discover new quantitative imaging biomarkers and integrate them in decision support tools (often powered by artificial intelligence) to aid radiologists in challenging diagnostic classifications [9]. The theory behind it is sound: microscopic heterogeneity in medical images relates to biological tumor heterogeneity, and might therefore deliver insights for tissue characterization [10]. Nevertheless, beyond hopes and hype, it is important to remember that the quality of studies on the applications of radiomics to adrenal imaging is heterogeneous and overall lower than desirable [11]. Radiomics is an undoubtably complex multistep technique, often paired with other complicated tools, such as machine learning and artificial intelligence, which means that several challenges need to be properly faced in order to deliver reliable results [12]. For instance, high-quality datasets are necessary to avoid the “garbage in garbage out” issue, rigorous handling of data is required to minimize the risk of data leakage during modeling, and an added benefit of radiomics over less complex and already-available techniques should be demonstrated. Overall, the greatest concern is in regard to the so-called reproducibility crisis, with uncertainty clouding the generalizability and clinical applicability of experimental evidence [13]; it remains to be seen whether radiomics-based solutions for adrenal mass characterization will eventually become available for radiologists in clinical practice.

In conclusion, and going back to the question in the title, it appears that some quantitative imaging approaches are already an established reality for adrenal mass characterization, while others can only be considered a possibility at present. Both are illustrated with startling clarity and succinctness in the review article by Barat and colleagues [4], a must-read for radiologists and physicians with a keen interest in adrenal imaging.
